# A Review of Two Decades of Conservation Efforts on Tigers, Co-Predators and Prey at the Junction of Three Global Biodiversity Hotspots in the Transboundary Far-Eastern Himalayan Landscape

**DOI:** 10.3390/ani11082365

**Published:** 2021-08-10

**Authors:** Mriganka Shekhar Sarkar, Diana Ethel Amonge, Nawraj Pradhan, Hla Naing, Zhipang Huang, Mahendra Singh Lodhi

**Affiliations:** 1 North-East Regional Centre, GB Pant National Institute of Himalayan Environment (GBPNIHE), Itanagar 791113, India; dianaamonge5@gmail.com (D.E.A.); mslodhi@gbpihed.nic.in (M.S.L.); 2 International Centre for Integrated Mountain Development (ICIMOD), GPO Box 3226, Kathmandu 44700, Nepal; Nawraj.Pradhan@icimod.org; 3 Wildlife Conservation Society Myanmar Program, No. 100, Yadanar Myaing Street, Ward (1), Kamayut Township, Yangon 11041, Myanmar; hnaing@wcs.org; 4 Institute of Eastern-Himalaya Biodiversity Research, Dali University, No. 2 Hongsheng Road, Dali 671003, China; huangzp@eastern-himalaya.cn

**Keywords:** large carnivores, Far-Eastern Himalaya, HI-LIFE, conservation, transboundary co-operation

## Abstract

**Simple Summary:**

The Far-Eastern Himalayan Landscape, a global biodiversity hotspot spread across parts of India, Myanmar, and China, holds great potential for the long-term conservation of tiger populations. National-level efforts aimed at tiger conservation, such as the creation of protected areas, have been critical for the survival of key tiger populations. However, for tigers to continue to survive into the future, it is important that these key populations remain connected with each other, particularly in this transboundary landscape. This requires greater regional and cross-border co-operation in conservation research, monitoring, and planning to protect habitats and corridors. Here, we review and synthesize the available literature on two decades of conservation efforts aimed at the study and conservation of tigers, their co-predators, and prey in the landscape to understand research trends, identify knowledge gaps, and suggest priority areas for future research and conservation interventions. This review could be useful for formulating conservation plans and actions that could help in the recovery of tiger populations; for identifying, restoring, and securing connectivity between key tiger habitats; and for addressing other key threats, such as habitat loss, poaching, and trade in wildlife parts.

**Abstract:**

Addressing the effects of human-caused habitat destruction on free-ranging threatened large carnivores requires actions that go ‘beyond borders’ in conserving and protecting their habitat and prey base. In this review, we compiled information from available literature on 20 years of conservation efforts aimed at tigers, co-predators, and their prey in the Far-Eastern Himalayan Landscape that is situated at the confluence of three global biodiversity hotspots covering parts of India, Myanmar, and China. The vast area of the proven biodiversity-rich forested landscape is highly suitable for long-term survival of carnivores, such as tigers. Habitat loss, ritual hunting, commercial exploitation, and poaching are the prevailing threats that have resulted in low tiger, co-predator, and prey population densities. Studies suggest that tiger presence is confined to a few areas, while other tiger populations have been extirpated across most parts of the landscape. Past research also suggests that the landscape holds low abundance of diverse prey species richness (*n* = 22), and urgent conservation measures are required to improve their habitat and numbers. This calls for greater regional and transboundary co-operation on research and knowledge sharing, conservation awareness programs for locals, and cross-border co-operation on wildlife monitoring. Strict policies are also required to enable PA managers to develop strategic plans to conserve large predators and protect their habitats and corridors.

## 1. Introduction

Large carnivores are categorized by their large body size and for being apex predators that occupy the top of the food chain [1]. The tiger (*Panthera tigris*), the most iconic large carnivore, and other sympatric predators, such as the leopard (*Panthera pardus*), clouded leopard (*Neofelis nebulosa*), and Asiatic wild dog (*Cuon alpinus*), are found in the forested ecosystems of southeast Asia [2]. Being keystone, flagship, and umbrella species, they play a crucial role in regulating and maintaining forest ecosystems, and their loss can have long-term impacts on ecosystem stability and processes [3]. Globally, many large carnivore populations are on the verge of collapse, which poses a serious risk of local and, in some cases, complete extinction [3].

Carnivores such as tigers require undisturbed habitats in a large landscape, especially with abundant prey [4], to uphold long-term genetic and demographic viability [5,6]. To protect and conserve such free-ranging large animals, the availability of several such tiger habitats in a large landscape that are connected with each other via corridors is a prior requirement for better sustainability [7]. Moreover, other studies in India and Nepal indicate that, as long as they have abundant prey, tigers can also persist well outside protected areas [8,9]. Thus, 76 important Tiger Conservation Landscapes (TCLs) have been identified across the globe based on the quality of habitat and their potential to boost conservation efforts. Of these, 29 TCLs (priority Tiger Conservation Landscapes, Tx2 sites) have adequate interconnected quality habitat to meet the population recovery goal of doubling the wild tiger population [7]. Many such TCLs, however, fall between two or more countries and lack detailed monitoring information, making it difficult to evaluate efficient tiger conservation strategies [10,11,12].

The Far-Eastern Himalayan Landscape (FeHL) is one such Tx2 TCL situated at the confluence of China, India, and Myanmar, and covers 28 eco-regions (Table S1, Supplementary Information—1). The landscape shares a large mosaic of contiguous and connected forests having maximum potential to double the tiger population in the wild [13]. This is also one of the most diverse landscapes from an ecological point of view in the Southeast Asian region where carnivores, especially tigers, are suffering from major anthropogenic threats, such as poaching and hunting [14,15,16,17].

The Landscape Initiative for Far Eastern Himalayas (HI-LIFE) was introduced for conservation and sustainable development within a part of this FeHL and designed through a consultative process for long-term co-operation and conservation [18]. This region includes parts of three global biodiversity hotspots, namely Himalayas, Highlands of Southwest China, and Indo-Burma [19]. The HI-LIFE approach includes field research, the identification of landscape changes (human induced), and facilitating transboundary co-operation to better inform and improve conservation efforts in the respective landscape parts within China, India, and Myanmar. The Indian portion, i.e., Arunachal Pradesh, has tiger populations in the Pakke–Nameri and Namdapha–Kamlang PA conservation complexes. Namdapha–Kamlang has a smaller tiger population compared to the Pakke–Nameri conservation unit [20]. There is also some evidence of tigers in the forests of Lower Subansiri, Changlang, East Kameng, and Tirap districts [20].

Namdapha Tiger Reserve in the Indian Eastern Himalayas contains the last large tracts of lowland Dipterocarp forests in Southeast Asia, and is designated as the world’s northernmost tropical rainforest, having the highest species richness among all Indian protected areas [17]. The Hukaung Valley TR in northern Myanmar is the largest tiger reserve in the world [21] and is located at the center of the FeHL region. The Namdapha TR, Hukaung Valley TR, and other adjoining wildlife sanctuaries together constitute a larger tiger landscape and hold high potential for tiger and sympatric or co-predator conservation [22]. Here, sympatric or co-predator refers to carnivore species that primarily obtain their food by hunting tiger prey species or prey of relatively smaller body size. However, due to low priority in research, rarely conducted (infrequent) conservation monitoring programs, and inaccessibility and remoteness of the landscape, very few studies have been conducted so far on the status of tigers, co-predators, and their potential prey species over the past two decades. The knowledge on their status is poorly documented, and that hinders managers and forest officers from formulating suitable management plans and long-term monitoring programs for carnivores in this unique and relatively pristine landscape. Moreover, impenetrable forests and heavy rainfall have ensured that the landscape has remained largely underexplored and was almost completely isolated until the middle of the 20th century. The British did not try to bring the highlands under their administrative control, as they were seen as unproductive [23], and this prevented commercial exploitation of forests. This could be a reason why this landscape is less fragmented in comparison to other TCLs [13].

At present, the landscape holds a few carnivore species (viz. tiger, leopard, wild dog, etc.) at low densities, but appropriate monitoring and conservation strategies could help large carnivore conservation in this region. The successful outcome of this initiative in this landscape can be replicated in the vast adjoining areas of Southeast Asia, having similar climatic and socioeconomic conditions. In this context, a 20-year review of conservation efforts focused on carnivores and their prey species in this landscape could serve as a starting point to develop monitoring programs, and prioritize research and strategic management. Here, we review and synthesize the existing and accessible peer-reviewed literature covering the status of large carnivores, including tigers, co-predators, and their potential prey species, in the landscape to understand the research trends, identify knowledge gaps, and suggest priority conservation areas for future research and management. This baseline literature review could provide important information for the formulation of conservation plans and actions that could help in the recovery of large carnivore populations and prevent their local extinction.

## 2. Materials and Methods

### 2.1. Study Area

**Figure 1:**
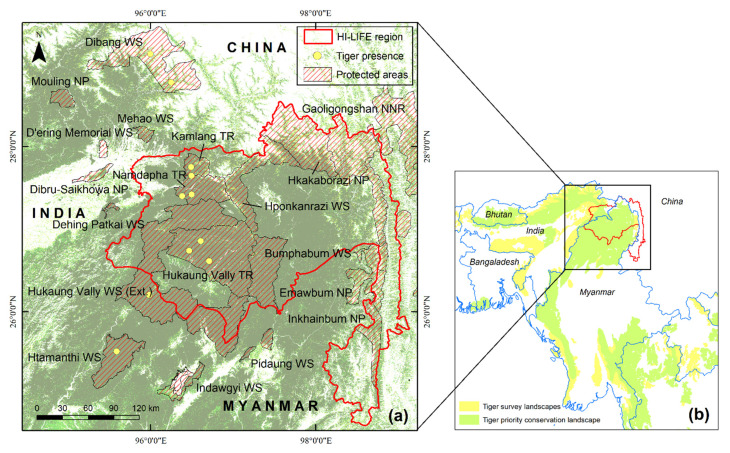
(**a**) The Far-Eastern Himalayan Landscape (FeHL) map depicts protected areas that include HI-LIFE working areas; (**b**) the priority tiger conservation landscape and survey landscape [26].

FeHL (24°37′40.09″–28°32′35.3″ N and 95°27′13.75″–99°8′15.57″ E) covers an area of more than 71,400 km^2^ and ranges in elevation from 200 to 5800 m a.s.l. [24]. It is a transboundary landscape that stretches from the Nujiang River and Gaoligongshan Nature Reserve of China to Namdapha Tiger Reserve, Kamlang Tiger Reserve, and Dibang Valley Wildlife Sanctuary of India in the east, and the Hkakaborazi National Park, the Hponkanrazi Wildlife Sanctuary, and Hukaung Valley Wildlife Sanctuary of Myanmar in the center (Figure 1). The governments of China, India and, Myanmar have supported HI-LIFE because of its regional and global importance, and have emphasized regional co-operation for the conservation and development of integrated landscapes [25].

### 2.2. Review Methods

We adopted the Search, Appraisal, Synthesis, and Analysis (SALSA) framework [27] by following Mengist et al. (2020) [28] to find the best available literature for this review. The review focused on global distributional range, status, and conservation efforts aimed at large carnivores and their potential prey species found within the HI-LIFE area (FeHL) and in the adjoining areas. The major purpose of the paper is to document the various research efforts and identify gaps that will need to be addressed in future interventions for the long-term monitoring and conservation of these predators, prey, and their habitats. Scopus and Google Scholar search engines were used with the names of each protected area as the primary key word, followed by a further filtering of the search results with other relevant keywords, such as mammal, animal, carnivore, hunting, tiger, leopard, snow leopard, clouded leopard, wild dog, etc. The search covered journal articles, books/ book chapters, master’s and Ph.D. dissertations, PA management plans, and institutional reports. We also considered scientific literature on carnivores, prey, and conservation issues at the global level that partially included HI-LIFE and the adjoining regions. The collected publications (*n* = 351) were further carefully searched for data and information specific to the status of carnivore species, their prey, human–carnivore conflict, and conservation issues. Based on this, 79 articles were selected as being relevant to the purpose of this review.

## 3. Results and Discussion

### 3.1. Status of Tigers and Co-Predators

The HI-LIFE region includes two tiger reserves (viz. Namdapha Tiger Reserve, Hukaung Valley Tiger Reserve), three adjoining wildlife sanctuaries (viz. Hponkanrazi Wildlife Sanctuary, Bumhpabum Wildlife Sanctuary), and two national parks (viz. Gaoligongshan National Nature Reserve, Hkakaborazi National Park) that together constitute a larger tract of connected habitats harboring large- to medium-sized carnivores, including the tiger (*Panthera tigris*), leopard (*Panthera pardus*), Asiatic wild dog (*Cuon alpinus*), clouded leopard (*Neofelis nebulosa*), and red fox (*Vulpes vulpes*) (Table 1).

In the extended Far-Eastern Himalayan Landscape (FeHL), tigers were found to inhabit Namdapha TR, Dibang WS, and Kamlang TR in the Indian part, and in Hukaung Valley TR, Htamanthi WS, and adjoining forest patches in northern Myanmar. Tigers and leopards were reported locally extinct in 1983 and 1985, respectively, within Gaoligongshan National Nature Reserve, China [39].

Historically, Namdapha TR had estimated that there were 49, 52, and 57 tigers during 1993, 1995, and 1997, respectively [40]. Much of the TR has dense vegetation cover, with high hills and numerous rivers and seasonal streams. The TR is also contiguous with northern Myanmar’s pristine habitats for tigers. Historically, tigers were found across northern Myanmar’s forests, although they were not estimated prior to 2002 [38]. Myanmar has a long history of tiger hunting [41,42,43,44]. Tigers were traditionally considered pests and, until 1931, the government provided licenses and rewards for killing them. This led to depopulation on a massive scale through sport hunting. For example, during a 4-year period from 1928–1932, 1382 tigers were reported killed in British Burma [45]. In 1999, WCS Myanmar, in collaboration with the Myanmar Forest Department, undertook a study to determine the current status and distribution of tigers to develop an updated national strategy for tiger conservation and management [38]. They estimated the presence of 7–71 tigers inside a 3250 km^2^ area of prime tiger habitat of Hukaung Valley Tiger Reserve [46]. Similarly, hunting was also prevalent in the Gaoligongshan region of Yunnan Province, leading to the extirpation of tigers from this region [47].

Just over a decade ago, a camera-trap-based survey during 2006 and 2007 identified no trace of tigers in Namdapha TR [36]. A systematic survey with 36 camera traps (trap night effort of 1725) during 2018 also did not yield any tiger images [30], although the presence of three individual tigers was confirmed from pug marks and scat-based DNA analysis [48]. However, tiger sightings by tourists and cattle kills by tigers have been reported from near the western boundary of the park. Tiger pug marks were also recorded on multiple occasions during opportunistic surveys [36]. As such wildlife officials believe that Namdapha TR (which varies in altitude from 200 to 4571 m a.s.l.) is the least explored forest area in the state because of its steep undulating terrain. They believe that the TR still harbors ideal tiger habitat and needs rigorous investigation [36]. Kamlang is the recently notified 50th Indian Tiger Reserve, situated north of Namdapha TR, and has recently confirmed tiger presence by scat-based DNA profiling [30]. Dibang Wildlife Sanctuary and the Mishmi Hill range are situated to the northwest of Namdapha TR, and are well connected through forest patches with consistent reports of tiger presence for many years [31,49]. In December 2012, two tiger cubs were rescued from Angrim Valley of Anini Tehsil, located at 1968 m a.s.l. in Dibang Valley WS [31]. During a study in Dibang Valley WS, at an altitude of 1765 m a.s.l., an adult tiger was photo-captured in the Chelo Pani Camp. Pug mark and scat evidence of tigers were also recorded at 2065 m a.s.l. in the Ange Pani area of Dibang Valley WS [31]. Subsequently, two independent camera-trap-based studies were carried out during 2014–2017 at the Dibang Valley WS; the first one reported the presence of nine adult tigers and two cubs [49], and the second recorded five tigers [50]. Moreover, camera trap surveys in 2018 also reported the presence of two unique adult tigers [30].

The southern part of Namdapha TR is connected to the forest tracts of Myanmar, which have much higher potential for harboring wild tigers. However, tigers are facing an even greater threat to their survival in this part of the landscape. Based on historical records, tigers were widespread throughout the country. However, recent surveys have found no evidence of tigers, except in four sites, which include Hukaung Valley TR and Htamanthi WS [51]. In fact, the Hukaung Valley TR is the largest known tiger habitat (17,373 km^2^) in the world. Between November 2002 and June 2004, tigers were recorded and identified by camera traps. Their density was also estimated through the photographic capture–recapture method. With the presence of 7–71 tigers, the density was estimated at between 0.2 and 2.2 tigers/100 km^2^ [21]. Another long-term camera-trap-based study reported consistent tiger presence from 2001 to 2011, with records of 35 other mammalian species (viz. 19 carnivores, 1 elephant, 4 primates, 1 pangolin, 6 even-toed ungulates, and 4 rodents). However, the study also found a steady decline in tigers during the sampling period [22]. Recently, during 2017–2018, the Forest Department spotted a male tiger in the western part of Hukaung Valley TR (between Namyun and Shinbweyan towns of Naga Hill, along the Ledo Road) during an opportunistic camera trap survey. The Htamanthi WS, situated to the southwestern side of Hukaung Valley Tiger Reserve, is a very promising source site for tigers, and falls within the southern part of the FeHL, where tiger monitoring has been conducted since 2014. Initially, five tigers (four female and one male) were detected in this region. Three years later, a breeding female tiger was spotted, which gave birth to three cubs (personal communication with H. Naing).

Moreover, two new male and female tigers migrated to Htamanthi WS and they have been consistently sighted for a few years [52,53]. The northern part of the Hukaung Valley TR is connected through long patches of forest to Hkakaborazi NP and Hponkanrazi WS that have no historical record of tigers or other large carnivores. However, the forest extends further into China’s Yunnan Province, up to the Gaoligongshan National Nature Reserve, which is part of the historical tiger range. Recent reports indicate that very few carnivores (except tigers) inhabit the region. In 1983, a tiger was killed by locals using poison near Guyong of Gaoligongshan National Nature Reserve because it killed some domestic animals. However, locals and researchers have so far found no other evidence of tiger presence. In another study, poachers interviewed agreed that, even if they were present, they would be very rare to sight [47]. More recent surveys have confirmed that it is not only tigers that are locally extinct in the region, but also the grey wolf (*Canis lupus*), red fox (*Vulpes vulpes*), raccoon dog (*Nyctereutes procyonoides*), Asiatic wild dog or dhole (*Cuon alpinus*), clouded leopard (*Neofelis nebulosa*), and leopard (*Panthera pardus*) [29]. The relative abundance of tigers and co-predators in the various protected areas of the FeHL is presented in Table 1, and their body weight classes in Table S2 (Supplementary Information—2).

### 3.2. Status of Potential Prey Species

Very few studies have been conducted in Namdapha TR to rigorously assess the distribution and abundance of potential prey species of large carnivores. A study by Datta et al. (2008) assessed prey status using camera traps at Namdapha TR (covering 30% of the reserve), where they only obtained cumulative 156 prey photographs [30]. Among them, large prey (sambar *Rus unicolor* and wild pig *Sus scrofa*) constituted only 3.9%. The rest of the pictures were of small prey, such as porcupine (*Hystrix indica*) and muntjacs (*Muntiacus muntjak*). Muntjacs alone constituted 45.5% of all prey species photo-captured. Recent camera-trap-based surveys at Namdapha TR have reported the presence of 25 mammal species, of which five species (viz. wild pig, *Sus scrofa*; Chinese serow, *Capricornis sumatraensis*; sambar, *Rus unicolor*; gaur, *Bos gaurus*; and Indian muntjac, *Muntiacus muntjak*) could be potential prey for tigers and other co-predators [30]. Kamlang TR is also reported to have 24 mammalian species, of which five (viz. Indian muntjac, *Muntiacus muntjak*; gaur, *Bos gaurus*; mithun, *Bos frontalis*; Chinese serow, *Capricornis sumatraensis*; and wild pig, *Sus scrofa*) could be potential prey for tigers and other co-predators [30]. The low photo-capture rate signifies very low-density prey populations, but this requires further investigation. Apart from the above studies, other research works have only reported the inventory of prey species available in other protected areas in the landscape [15,16,22,29,32,38] (Table 1). Five deer species were recorded from the Gaoligongshan NNR. They are sambar (*Rus unicolor*), red or Indian muntjac (*Muntiacus muntjac*), tufted deer (*Elaphodus cephalophus*), Gongshan muntjac (*M. gongshanensis*), and hog deer (*Axis porcinus*). Indian muntjac and the tufted deer were the most common and most widespread in the Gaoligongshan NNR. Interestingly, the Gongshan muntjac was first discovered and described in 1988 and 1990, respectively, through the karyotype method [54,55]. The previously recorded hog deer from Ruili County in the south has now probably disappeared. Moreover, gaur (*Bos gaurus*) is historically known from the southern part of Gaoligangshan, but is now extirpated from most of the lowland areas; some remnant populations are sparsely distributed in mountainous areas, and others have crossed the Myanmar border. Takin (*Budorcas taxicolor*) was once widespread throughout Gaoligongshan (their southernmost extent). Although they are still relatively abundant along the core region of the Gaoligongshan Range, they have virtually been extirpated in the rest of the region. The red goral (*Naemorhedus cranbrooki*) is known only from the northern highlands of Gaoligangshan [55]. Their numbers seem to be quite small, and their hunting is still out of control. So, the future of this population looks bleak [55]. Himalayan goral (*Naemorhedus goral*) and blue sheep (*Pseudois nayaur*) are distributed in the extreme north of Gaoligongshan, where both are found in very small numbers [47]. A recent study reported that the leaf muntjac (*Muntiacus putaoensis*) is locally extinct [29]. The relative abundance of selected potential prey species is listed in Table 1, and their body weight classes presented in Table S1.

### 3.3. Hunting and Other Anthropogenic Disturbance

The Indian part of FeHL has 26 major tribal communities and over 100 different tribal subgroups [23]. Myanmar’s Hukaung Valley TR has five major ethnic groups. The Singpho and Naga tribal groups reside in the northern part of the valley, the Lisu along the northern boundary, and the Shan and Myanmar in the central dry zone [51]. Most tribes in Namdapha TR and Hukaung Valley TR are involved in forest-based subsistence and commercial activities, such as slash-and-burn agriculture, harvesting of cane, fishing, cardamom cultivation, timber extraction, and gold mining. These livelihood activities are detrimental to the main species, tigers, and their co-predators and prey [51]. A similar situation is seen on the Chinese side of the FeHL region, where the area is dominated by the Lisu tribe and extraction of natural resources, hunting for bush meat, and medicinal plant extraction from mountain areas is dominated by them [29]. Although wild/bush meat plays a small role in household consumption of the above tribes, it contributes significantly to household income. Thus, the local people’s dependence on hunting to address their socioeconomic needs has already reduced both large carnivores and their prey base within the landscape [15,16]. For example, past research suggests that very few tigers and other large predators are left in Namdapha TR, and even larger prey, such as sambar and wild boar, are rarely sighted [17,36]. A similar condition is observed in the Myanmar [46] and China [29] parts of the landscape.

Therefore, hunting of wildlife for ritual purposes, illegal trade, and food is widespread in the HI-LIFE area and the extended FeHL landscape, and is prevalent among most tribal groups living in this landscape [36,56,57,58]. Dried fish and wild meat are sold in the nearby villages [56,59]. Other anthropogenic threats to wildlife and habitat include expansion of agriculture, slash-and-burn cultivation, establishment of tea gardens and coffee estates, development projects, and illegal trade in animal body parts for traditional medicine [15,22,32,36,56]. The skins and skulls gathered by villagers residing around Namdapha TR suggest that approximately 34 species of mammals have been hunted [56].

The main target species for hunting in this region are primates and ungulates. Musk deer (*Moschus fuscus*), bear sp., and otters (*Lutra lutra*) are also commonly killed [17]. Elephants have almost disappeared from this region [17]. There is also a report of at least 15 tigers hunted during the period from 1994 to 2003 in Namdapha [17]. There are also records of illegal hunting of tiger (*Panthera tigris*), musk deer (*Moschus fuscus*), elephant (*Elephas maximus indicus*), bear sp., otters (*Lutra lutra*), and other small cats [56,59]. Compared to Namdapha TR and Kamlang TR, the Dibang Wildlife Sanctuary is of importance for the conservation of unique and genetically distinct species of carnivores. Here, local beliefs are supportive of tiger conservation, as the local aboriginal community considers the tiger to be their elder brother [60].

In the Indian part of the FeHL, hunting is usually done with guns, crossbows, and a variety of local-made traps, and metal leg traps are used for tigers [36]. Road widening and expansion can lead to easier access for poachers and hunters [61]. For example, a 157 km road was built in 1972, extending from Miao to Vijoynagar, that crosses the core habitat of the park [36]. The former road was only about 16 km within the park, but, during 2010 and 2011, the Government of Arunachal Pradesh widened it till Vijoynagar (near the Myanmar border) to connect a population of about 6,000 Lisu and Nepalis living in the area. On the earlier road, sambar (*Rus unicolor*) and indian muntjac (*Muntiacus muntjak*) were often seen eating fallen fruit at night and basking during the day. As the road became wider, many locals reported a decrease in sambar sightings along the road. Its root cause may be poaching or hunting [61].

The Hukaung Valley Tiger Reserve was first denoted as a high priority conservation site within Myanmar after a cumulative assessment carried out by Myanmar Forest Department, Wildlife Conservation Society, and international scientists in 1999 [51]. However, the practice of illegal hunting and poaching is still prevalent in this region. For example, poachers (viz. persons carrying hunting/fishing gear, gun, snare, spear, single-action rifle, shotgun, homemade gun, blanket or cloth for making a hide, fishing net, ring net, fishing rod, electro-fishing equipment, poison, bow and arrow) and villagers with poaching tools were frequently photo-captured on camera traps installed throughout the region for wildlife census during 2001–2011 [14,22]. Local communities, particularly the poorest households within Hukaung Valley TR, benefit from work opportunities offered by gold mining [62] and rattan trade [63]. These activities could be a secondary but major cause for wildlife loss in this part of the region by causing huge disturbance to their natural habitat.

In the Hponkanrazi Wildlife Sanctuary, some of the most commercially valuable species preferred by local hunters were either completely absent from hunting records (such as tigers, otters, and musk deer) or were rarely collected during actual hunting (such as pangolins and bears) [15]. Species obtained from hunters are generally commercially valuable species (for example, muntjac), whereas species that have low commercial value are not chosen by hunters [15,32]. No poaching cases were recorded in Bumhpabum Wildlife Sanctuary, as the sighting of wild animals has been rare in this region. Serow (*Capricornis milneedwardsi*), red goral (*Naemorhedus baileyi*), muntjac (*Muntiacus* sp.), bear sp. (*Ursus thibetanus/Helarctos malayanus*), Assamese macaque (*Macaca assamensis*), black musk deer (*Moschus fuscus*), and takin (*Budorcas taxicolor*) are the species commonly targeted by hunters, accounting for about 90% of the wildlife trade around Hkakaborazi National Park [16]. Commercially valuable species, such as tigers (*Panthera tigris*), Eurasian otter (*Lutra lutra*), and Chinese pangolin (*Manis pentadactyla*), previously targeted by hunters are completely missing from current records of hunting, indicating that they are now absent in the wilderness [16]. Although agriculture is a major occupation, hunting (driven by trade) represents a significant source of income for the locals compared to other major livelihood activities [16].

The Gaoligongshan National Nature Reserve is situated within the Gaoligong Mountains of western Yunnan Province of China. The region is predominated by Lisu tribes. Prior to 1950, their livelihood depended heavily on hunting, and offerings were made to the hunting god before each hunting expedition, believing that the god would ensure their safety and bless them with a good harvest. However, currently, villagers have limited their main livelihoods to agriculture due to the recent austerity measures [64]. Lisus have a long history of “slash and burn” farming to meet their main livelihood needs. In addition, there are issues of illegal hunting, poaching, and the indiscriminate collection of resources for traditional medicine [29]. Asiatic black bear (*Ursus thibetanus*) remains widespread and common, but other major large carnivores in this area have disappeared. There is negative human–bear interactions resulting in casualties on both sides annually [46]. Major prey species, such as musk deer (*Moschus fuscus*), tufted deer (*Elaphodus cephalophus*), Gongshan muntjac (*Muntiacus gongshanensis*), Burmese red serow (*Capricornis rubidus*), and sambar (*Rus unicolor*), are rarely seen in the reserve [29]. The killing of various primate species for food is a ritual practice. Humans appear as the main predators of hoolock gibbons (*Hoolock hoolock*) and other primate species throughout southwest China [65,66], and hunters can find them by tracking their loud calls.

### 3.4. Past and Ongoing Conservation Initiatives

In the Indian part of the FeHL, the Namdapha TR and Kamlang TR falls under the umbrella of Project Tiger, which is a national tiger conservation program launched in April 1973 by the Government of India. Other than routine monitoring of wildlife habitat by the Forest Department through annual short-term camera trapping and scat DNA-based individual identification of tigers [30,50,67], rigorous long-term research on carnivores and their unique habitats in the Indian part of the FeHL (viz. Namdapha TR, Kamlang TR, Dibang Valley WS, and adjoining forested area) remain inadequate in comparison to peninsular India for the last 20 years. A few inventory-based studies have been conducted on carnivores and their prey species, such as the search for new highland tiger habitat in the vicinity of Dibang Valley WS [31,49] and wildlife assessment through camera traps within Namdapha TR [36], with financial support from the National Tiger Conservation Authority and local forest department. Few sporadic conservation education programs have also been conducted. US Fish and Wildlife Service sponsored a project on “Conservation education and capacity building on tiger conservation in the protected areas of Arunachal Pradesh” to provide conservation education training to local teachers, NGOs, and volunteers. They also conducted capacity-building training for forest personnel. Zoo Outreach Organization (ZOO) of Coimbatore, Tamil Nadu executed the training with the support and co-ordination of Namdapha TR, and conducted several teacher training workshops for tiger conservation, named “Teachers for Tigers” [68,69].

In 1997, the Myanmar government formally requested that the Wildlife Conservation Society (WCS) develop a tiger conservation plan for the entire country, where Hukaung Valley Tiger Reserve was included [70]. During 1981, the Myanmar government estimated the number of tigers in the country at 3000 [70]. However, there is evidence that tigers were missing from many of their former ranges [44] and threatened in their existing ranges [71]. This suggests the need for a revised conservation assessment. WCS has been co-operating with the Myanmar Forest Department since 1994 under a Memorandum of Understanding. In 1999, a project was launched to determine the current habitats of tigers across the country and to define the various management activities required for the conservation of tigers in their natural habitat, especially in the Hukaung Valley TR [70]. The National Tiger Survey (1998–2002) identified high potential tiger conservation areas in Myanmar, where Hukaung Valley Tiger Reserve was established. Panthera’s Tigers Forever Program (2006) Fund explored tigers, leopards, and dhole of the region through scat-based genetic survey. WildCRU’s clouded leopard (2014–present) grant has explored the presence of tigers in Htamanthi WS. IUCN KfW (2015–2019), Segre/Instituto Oikos’s sun bear (2015–2019), and USFWS’s tiger (2018–2019) grants have supported regular monitoring of tigers in the region.

Large carnivores became locally extinct long ago in the Chinese portion of the FeHL [29]. However, monitoring and species-specific research is still ongoing in the area, especially on takin, red serow, capped langur, and Skywalker hoolock gibbon (Li et al. unpublished). In 2015, two studies on takin were conducted in the Dulongjiang area of the Gaoligongshan Mountains, the first on habitat use and the second on foraging habits.

Another takin conservation and monitoring program was conducted in Gaoligongshan Nature Reserve (Tengchong) during 2016 and 2018, where researchers collected video evidence of takin, investigated the population size, and conducted public awareness activities around the reserve. Another study was launched in 2019 on a regional inventory of red serow in Gongshan County, which revealed their distribution in 58 locations of the county, including 31 locations within the protected area and 27 locations outside the protected area. Two separate studies on capped langur and Skywalker hoolock gibbons were conducted at the Gaoligangshan Mountains (2018–2019) and Gaoligongshan Nature Reserve (2016–2020), respectively, where each study provided data on the habitat distribution of the species and, in turn, helped establish long-term monitoring of these species (personal communication with H. Zhipang).

## 4. Conclusions and Management Recommendations

In this paper, we have attempted to review conservation efforts and the status of tigers, co-predators, and their prey species in the FeHL over the past 20 years. However, due to the very small number of studies in this little explored landscape, we were unable to present any statistically significant results. It is clear that research on carnivores and their prey is needed in this landscape. However, through this review, we have been able to shed light on trends in the conservation status of tigers, co-predators, and prey species and the conservation challenges in this transboundary landscape. The following section contains some concluding remarks, along with conservation management recommendations that could strengthen transboundary conservation and research efforts in the future.

(i) Tiger and large co-predator abundance are mostly influenced by prey availability and the existence of anthropogenically undisturbed habitats. Past studies have suggested that the FeHL has high potential to harbor tigers and large co-predators because of the high connectivity among dense forest patches between India, Myanmar, and China [13].
(ii) Effective awareness building programs and alternative livelihood opportunities for local communities are needed to reduce pressure on forests and biodiversity. There is a need to address the issue of hunting by employing local communities in the management of protected areas and buffer zones; enhancing the technical capacity of protected area workers; implementing an integrated land-use plan aimed at stabilizing land use; and amending existing wildlife laws to comply with obligations under various international treaties, such as the Convention on International Trade in Endangered Species of Wild Fauna and Flora (CITES) [72]. Improved leadership training for relatively higher-level forest officials, such as Sub-divisional Officers (SDOs) and range officers, can also improve regional park management.
(iii) Long-term wildlife monitoring is essential not only to know the status of target species, but also to assess the efficacy of the socioeconomic interventions in bringing about changes in resource dependence and helping wildlife recovery. The landscape still holds rich biodiversity, and large predators and their prey base can be restored if regional protected area authorities of the HI-LIFE area work with local communities. Wildlife authorities or park managers can think of innovative solutions by using local people’s skills and knowledge of the landscape for wildlife documentation and conservation, thereby giving them incentives to support, be involved in, and benefit from conservation efforts.
(iv) Community relations and funding for more integrated management of parks and people can reduce conflict, while more environmental education and outreach activities for local students can provide some much-needed services and create conservation stewards among the younger generation. Creative and sustainable ways of supporting all protected areas of HI-LIFE are needed. Partnerships with specialized government and nongovernment organizations can supplement traditional means of support [73]. For example, the Regional Forest Department should introduce specially designed agroforestry and social forestry programs to suit the needs of the local people in and around the park.
  Forest roads are important for the management of PAs within the HI-LIFE area and for communities living within and on the periphery of these forests. They are also necessary for accessing forestry resources [74]. They are also useful recreational features in a forest for tourists that allow for sightseeing and observing wildlife [75]. Although roads act as a great threat to most elusive wild animals and arboreal species [74,76], carefully constructed narrow forest trails or mud roads primarily help in routine patrolling and monitoring of wild animals in most protected areas, thus helping curb illegal activities, such as hunting and poaching, which are the major threat to carnivores and large-bodied prey species in this landscape [61]. Moreover, the construction of networks of forest trails and roads will also help researchers reach core areas within these PAs to undertake intensive sampling for estimating wildlife abundance and density with greater precision.
(v) The data from past research during 2008–2009 [21,36] show that the FeHL had good quality tiger habitats at Namdapha TR (India), Kamlang TR (India), Dibang WS (India), and Hukaung Valley TR (Myanmar), Htamanthi WS, and the adjoining forested regions. These tiger habitats are also connected to four other protected areas covering the eastern and northern parts of the Chindwin River that are minor sink habitats. These protected areas (from south to north) are Mahamyaing WS, Ya Baw Mee Key Biodiversity Area, Bumhpabum WS, Hponkanrazi WS, and Hkakaborazi NP. Together, they still have high potential as a landscape-level tiger metapopulation conservation unit in Southeast Asia. Extending the boundaries of Hkakaborazi NP to the south and west, linking it to the Hponkanrazi WS and/or adding sanctuaries to the Naung Mung region could probably further strengthen the natural habitat of tigers and co-predators. More studies are required to validate habitat structure, quality, and composition in these units in the FeHL.
(vi) There is an evolutionary significance of the tiger population in this landscape. The gene pool of tigers living in the FeHL matches the Indo-Chinese tiger (*Panthera tigris corbetti*) and represents a possible entry point for tigers in the Indian subcontinent [10]. The FeHL is also categorized as a Level I Tiger Conservation Unit and considered as a Priority Tiger Conservation Landscape [77]. All protected areas of the FeHL contain unique ecosystems and have a variety of habitats that include grasslands, tropical deciduous forests, alpine grassland, and snow-covered mountain tops in the north [10]. Other than protected tiger habitats, such as Namdapha TR, Kamlang TR and Hukaung Valley TR, the FeHL harbors many potential tiger habitats, including Dibang Valley WS, Htamanthi WS, and the adjoining fragmented forests, which together constitute a large tiger landscape connected through dense forested corridors. These tiger-holding forests outside protected areas need urgent conservation attention not only for tigers, but also for other co-predators to disperse and move. However, the future of these tiger and co-predator populations in the FeHL depends on appropriate management of the ever-fragmenting habitats and maintaining the existing populations as transboundary metapopulations. The reviewed literature [21,30,31] has indicated existing connecting corridors and pointed out critical zones (viz. within and outside tiger reserves) of FeHL where immediate conservation attention is needed. Given its enormous significance, the development of transboundary tiger and co-predator conservation corridors can be considered. The HI-LIFE landscape approach, which is transboundary in nature, can play an effective role in facilitating collaborative efforts between three countries and practical solutions to address the tiger conservation issues in the region [78].
(vii) Adequate human resources and funding for conservation action research are essential for the effective management and long-term monitoring of tigers and other large carnivores in this landscape. More importantly, for any management intervention, resource management needs to be strengthened through developmental research, pilot demonstrations, and information and data-sharing platforms at both a local and regional (landscape) scale [79]. These future interventions may hasten the generic understanding of the knowledge gaps and priorities for tiger and co-predator conservation in this unique landscape.
(viii) The future of large carnivores in the landscape lies in developing novel approaches for conservation in a multiuse landscape, which includes PAs and a variety of other land uses [80]. There are very limited data on tiger movement inside and outside of PAs, let alone transboundary movement that validates habitat connectivity. We also do not know how changes in climatic parameters (rainfall and temperature) might impact the ecosystems, including vegetation phenology [81]. Regional and transboundary co-operation, landscape-scale connectivity mapping, and characterizing the population genetics and gene flow of tigers are urgently required for PA managers and policy makers to develop strategic plans for conservation of tigers, co-predators, and prey species in this unique landscape. Finally, regional efforts and a transboundary conservation approach are much needed to conserve carnivores in this remote area of the FeHL.

## Figures and Tables

**Table 1:** Status of large carnivores and their potential prey species within important selected protected areas of the Far-Eastern Himalayan Landscape (data from the most recent studies).

	China	India			Myanmar			
Protected area name (IUCN Category)	Gaoligongshan National Nature Reserve (V)	Namdapha Tiger Reserve (II)	Dibang WS (IV)	Kamlang Tiger Reserve (IV)	Hkakabo razi National Park (II)	Hponkanrazi Wildlife Sanctuary (IV)	Hukaung Valley Wildlife Sanctuary/Extension (IV)	Htamanthi Wildlife Sanctuary (IV)
Reference	Li et al. 2019 [29]	Jhala et al. 2020 [30]	Gopi et al. 2014 [31]	Jhala et al. 2020 [30]	Rao et al. 2005 [32]	Rao et al. 2010 [15]	Naing et al. 2015 [14]	Naing et al. 2019 [33]
Survey method	Camera trap	Camera trap	Sign survey	Camera trap	Camera trap	Camera trap	Camera trap	Camera trap
Relative abundance index	Independent images/1000 trap-days	Number of trap-days/photo capture (trap-days: 1725)	Encounter rate/km	Number of trap-days/photo capture (trap-days: 573)	Capture rate per 100 trap nights (trap-days: 1238)	-	Number of ’independent photos/100 trap nights (trap-days: 7452 in core/3298 outside)	Capture rate per 100 trap nights (Catchment 1^β^: 7354 trap-days; Catchment 2^β^: 7192 trap days)
Large predators								
Tiger (*Panthera tigris*)	×	*, α	α, 0.38	α (CT-capture)	×		0.21/0.06	27; 3
Leopard (*Panthera pardus*)	×	157	0.08				0.01/0.00	0; 1
Wild dog (*Cuon alpinus*)	α	34	0.15		3.39	*	0.44/0.30	α
Clouded leopard (*Neofelis nebulosa*)	α	288		573	6.21	*	0.51/0.36	49; 54
Red fox (*Vulpes vulpes*)	α				α			
Grey wolf (*Canis lupus*)	α							
Prey species								
Indian muntjac (*Muntiacus muntjak*)		9	0.08	32	18.08	*	4.98/5.31	
Black muntjac (*Muntiacus crinifrons*)						*		
Leaf muntjac (*Muntiacus putaoensis*)	α	α	α	α	1.13	*		
Gongshan muntjac (*Muntiacus gongshanensis*)	0.20	α	α	α				
Northern red muntjac (*Muntiacus vaginalis*)	16.34							563; 491
Sambar deer (*Rus unicolor*)	*	24			*		1.60/2.82	0; 1
Hog deer (*Axis porcinus*)		α					0.19/0.00	
Musk deer (*Moschus fuscus*)		α			α	*		
Forest musk deer (*Moschus berezovskii*)	1.22							
Tufted deer (*Elaphodus cephalophus*)	3.65							
Wild pig (*Sus scrofa*)	3.38	41	0.06	143	10.73	*	0.98/0.94	141; 122
Gaur (*Bos gaurus*)		288		115		*	0.56/0.64	67; 12
Burmese red serow (*Capricornis rubidus*)	0.27					*	0.01/0.33	*
Chinese serow (*Capricornis sumatraensis*)	2.3	246	0.06	191	5.08			2; 0
Mishmi takin (*Budorcas taxicolor*)	*	α	0.08		α	*		
Red goral (*Naemorhedus baileyi*)		α			α	*		
Chinese goral (*Naemorhedus griseus*)	1.62							
Asiatic brush-tailed porcupine (*Atherurus macrourus*)	8.51	288		57	3.39	*	0.60/0.49	*
Himalayan crestless porcupine (*Hystrix brachyura*)	18.29	40		573	12.43	*	1.64/0.79	121; 138
Blue sheep (*Pseudois nayaur*)					α			
Mithun (*Bos frontalis*)				22				
Himalayan goral (*Naemorhedus goral*)			0.10					

Note: *-marked species were documented in recent studies, but relative abundance of that species was not calculated; ×-marked species were reported as locally extinct; α indicates presence of species reported in other past studies (China: Xue 1995 ^α^ [34]; Wang 2003 ^α^ [35]; and Chen and Qu 2010 ^×^ [29]; India: Datta et al. 2008 ^α^ [36]; Choudhury 2009 ^α^ [37]; personal communication from Kamlang Forest Department; Myanmar: Rao et al. 2011 ^α^ [16]; Naing 2015 ^α^ [22]; monitoring data by Naing et al. since 2014 ^α^; assessment by Panthera Foundation and Myanmar Forest Department ^α^; and Myanmar Forest Department 2003 ^×^ [38]; ^β^ camera trapping was conducted in two places of Htamanthi Wildlife Sanctuary).

## Data Availability

All data are presented in the manuscript.

## Supplementary Materials

The following are available online at https://www.mdpi.com/article/10.3390/ani11082365/s1, Table S1: Terrestrial Ecoregions of the World (TEOW) of Far Eastern Himalayan Landscape; Table S2: Potential prey and predators and their body weights of FeHL.
