# Supplementary material for: A Review of Two Decades of Conservation Efforts on Tigers, Co-Predators and Prey at the Junction of Three Global Biodiversity Hotspots in the Transboundary Far-Eastern Himalayan Landscape
Source: Animals (Basel). 2021 Aug 10;11(8):2365. doi: 10.3390/ani11082365 (PMC8388695; doi:10.3390/ani11082365)
Supplement: Supplementary file 1 [file animals-11-02365-s001.zip › Table S1_Supplementary Information 1.pdf]

animals MDPI

Table S1. Terrestrial Ecoregions of the World (TEOW) of Far Eastern Himalayan Landscape.

Sl No.	Ecoregions
1	Eastern Himalayan subalpine conifer forests
2	Northeast India-Myanmar pine forests
3	Eastern Himalayan subalpine conifer forests
4	Eastern Himalayan subalpine conifer forests
5	Eastern Himalayan alpine shrub and meadows
6	Eastern Himalayan broadleaf forests
7	Eastern Himalayan alpine shrub and meadows
8	Eastern Himalayan alpine shrub and meadows
9	Southeast Tibet shrublands and meadows
10	Nujiang Langcang Gorge alpine conifer and mixed forests
11	Hengduan Mountains subalpine conifer forests
12	Southeast Tibet shrublands and meadows
13	North-eastern Himalayan subalpine conifer forests
14	Eastern Himalayan alpine shrub and meadows
15	Eastern Himalayan alpine shrub and meadows
16	Eastern Himalayan alpine shrub and meadows
17	North-eastern Himalayan subalpine conifer forests
18	Eastern Himalayan subalpine conifer forests
19	Yunnan Plateau subtropical evergreen forests
20	Eastern Himalayan alpine shrub and meadows
21	Eastern Himalayan alpine shrub and meadows
22	Brahmaputra Valley semi-evergreen forests
23	Northern Triangle temperate forests
24	Northern Triangle subtropical forests
25	Northern Indochina subtropical forests
26	Mizoram-Manipur-Kachin Rainforests
27	Irrawaddy moist deciduous forests
28	Irrawaddy dry forests

Reference:

Olson, D. M.; Dinerstein, E.; Wikramanayake, E. D.; Burgess, N. D.; Powell, G. V. N.; Underwood, E. C.; D'Amico, J. A.; Itoua, I.; Strand, H. E.; Morrison, J. C.; et al. Terrestrial ecoregions of the world: a new map of life on Earth. *Bioscience*, **2001**, *51*, 933–938.
