# Supplementary material for: A Review of Two Decades of Conservation Efforts on Tigers, Co-Predators and Prey at the Junction of Three Global Biodiversity Hotspots in the Transboundary Far-Eastern Himalayan Landscape
Source: Animals (Basel). 2021 Aug 10;11(8):2365. doi: 10.3390/ani11082365 (PMC8388695; doi:10.3390/ani11082365)
Supplement: Supplementary file 1 [file animals-11-02365-s001.zip › Table S2_Supplementary Information 2.pdf]

animals

MDPI

Table S2. Potential prey and predators and their body weights of FeHL.

Sl. No.	Species type	Species common name (Scientific name)	Body weight
[1]	Large carnivore	Tiger (*Panthera tigris* (Linnaeus, 1758))	M: 220 to 260 kg.; F: 130 to 160 kg. (1)
[2]	Large carnivore	Leopard (*Panthera pardus* (Linnaeus, 1758))	M: 50 and 77 kg; F: 29 and 34 kg (2)
[3]	carnivore	Wild dog (*Cuon alpinus* (Pallas, 1811))	M: 15 to 21 kg; F: 10 to 17 kg (3)
[4]	carnivore	Clouded leopard (*Neofelis nebulosa* (Griffith, 1821))	M: up to 25 kg; F: 15 to 17 kg (4)
[5]	carnivore	Red fox (*Vulpes vulpes* (Linnaeus, 1758))	5–7 kg (5.1)
[6]	Prey species	Indian muntjac (*Muntiacus muntjak* (Zimmermann, 1780))	14 to 35 kg (6)
[7]	Prey species	Black muntjac (*Muntiacus crinifrons* (Sclater, 1885))	21.3 to 36.1 kg (7)
[8]	Prey species	Leaf muntjac (*Muntiacus putaoensis* Amato, Egan & Rabinowitz, 1999)	M: 12.1 kg; F: 11.8 kg (8)
[9]	Prey species	Gongshan muntjac (*Muntiacus gongshanensis* Ma in Ma, Wang & Shi, 1990)	18 to 24 kg (9)
[10]	Prey species	Northern red muntjac (*Muntiacus vaginalis* (Boddaert, 1785))	17 to 14 kg (10)
[11]	Prey species	Sambar deer (*Rusa unicolor* (Kerr, 1792))	M:185 to 260 kg; F: 162 kg (11)
[12]	Prey species	Hog deer (*Axis porcinus* (Zimmermann, 1780))	M: 40-55 kg; F: 30-40 kg (12)
[13]	Prey species	Musk deer (*Moschus fuscus* Li, 1981)	10 to 15 kg (13)
[14]	Prey species	Forest musk deer (*Moschus berezovskii* Flerov, 1929)	12 kg (14)
[15]	Prey species	Tufted deer (*Elaphodus cephalophus* Milne-Edwards, 1872)	17 to 50 kg (15)
[16]	Prey species	Wild pig (*Sus scrofa* Linnaeus, 1758)	66 to 272 kg (16)
[17]	Prey species	Gaur (*Bos gaurus* C.H. Smith, 1827)	650 to 1000 kg (17)
[18]	Prey species	Mainland Serow (*Capricornis sumatraensis* (Bechstein, 1799))	30 to 45 kg (18)
[19]	Prey species	Burmese red serow (*Capricornis rubidus* Blyth, 1863)	110 to 160 kg (19)
[20]	Prey species	Mishmi takin (*Budorcas taxicolor* Hodgson, 1850)	150 to 350 kg (21)
[21]	Prey species	Red goral (*Naemorhedus baileyi* Pocock, 1914)	20 to 30 kg (22)
[22]	Prey species	Chinese goral (*Naemorhedus griseus* Milne-Edwards, 1871)	20 to 30 kg (23)
[23]	Prey species	Asiatic brush-tailed Porcupine (*Atherurus macrourus* (Linnaeus, 1758))	1 to 4.3 kg (24)
[24]	Prey species	Himalayan crestless porcupine (*Hystrix brachyuran* Linnaeus, 1758)	0.7 kg-2.4 kg (25)
[25]	Prey species	Blue sheep (*Pseudois nayaur* (Hodgson, 1833))	35 kg-75 kg (26)

Reference for Table S1:

1. Choudhary, V. (2020). http://natureconservation.in/bengal-tiger-panthera-tigris-tigris-complete-detail-updated/
2. https://en.wikipedia.org/wiki/Indian_leopard
3. https://en.wikipedia.org/wiki/Dhole
4. https://www.aboutanimals.com/mammal/clouded-leopard/
5. Choudhary, V. (2020). http://natureconservation.in/snow-leopard-panthera-uncia-complete-detail-updated/
6. https://animaldiversity.org/accounts/Muntiacus_muntjak/
7. https://animaldiversity.org/accounts/Muntiacus_crinifrons/
8. Rabinowitz, A., Myint, T., Khaing, S.T. and Rabinowitz, S. (1999). Description of the leaf deer (*Muntiacus putaoensis*), a new species of muntjac from northern Myanmar. Journal of Zoology, **2006**, *249*,427-435**.** https://doi.org/10.1111/j.1469-7998.1999.tb01212.x
9. https://animaldiversity.org/accounts/Muntiacus_gongshanensis/
10. McShea, W. (2018). *Guide to the wildlife of Southwest China*. Smithsonian Institution.
11. https://animaldiversity.org/accounts/Rusa_unicolor/
12. https://indiabiodiversity.org/species/show/257368
13. https://animaldiversity.org/accounts/Moschus_fuscus/
14. https://thewebsiteofeverything.com/animals/mammals/Artiodactyla/Moschidae/Moschus/Moschus-berezovskii.html
15. https://animaldiversity.org/accounts/Elaphodus_cephalophus/
16. https://animaldiversity.org/accounts/Sus_scrofa/
17. https://animaldiversity.org/accounts/Bos_frontalis/
18. https://animaldiversity.org/accounts/Capricornis_sumatraensis/
19. http://www.ultimateungulate.com/Artiodactyla/Capricornis_rubidus.html
20. https://www.ecologyasia.com/verts/mammals/chinese-serow.htm
21. http://www.wilddocu.de/mishmi-takin-budorcas-taxicolor/ (see details in) Wilson, D. E. and Mittermeier, R. A. [eds], 2011: Handbook of the mammals of the world. Vol. 2. Hoofed mammals. Lynx Edicions, Barcelona.
22. http://www.wilddocu.de/red-goral-nemorhaedus-baileyi/
23. http://www.wilddocu.de/chinese-goral-nemorhaedus-griseus/ (see details in) Wilson, D.E. and Mittermeier, R.A. [Eds.]. (2011). Handbook of the Mammals of the World. Vol. 2. Hoofed Mammals. Lynx Edicions, Barcelona.
24. https://animaldiversity.org/accounts/Atherurus_macrourus/
25. https://en.wikipedia.org/wiki/Malayan_porcupine
